# Impact of capitation on physicians’ behavior among patients with hypertension: an interrupted time series study in rural China

**DOI:** 10.1186/s12889-024-18411-2

**Published:** 2024-05-03

**Authors:** Jiani Zhang, Jincao Yan, Yunke Shi, Ning Zhang

**Affiliations:** 1 School of Public Health, Capital Medical University, Beijing, 100069 P.R. China; 2 Chuiyangliu Hospital affiliated to Tsinghua University, Beijing, 100022 P.R. China

**Keywords:** Capitation, Integrated healthcare delivery system, Physicians’ behavior, Hypertension, Interrupted time series

## Abstract

**Objective:**

The purpose of this study is to explore the change in physicians’ hypertension treatment behavior before and after the reform of the capitation in county medical community.

**Methods:**

Spanning from January 2014 to December 2019, monthly data of outpatient and inpatient were gathered before and after the implementation of the reform in April 2015. We employed interrupted time series analysis method to scrutinize the instantaneous level and slope changes in the indicators associated with physicians’ behavior.

**Results:**

Several indicators related to physicians’ behavior demonstrated enhancement. After the reform, medical cost per visit for inpatient exhibited a reverse trajectory (-53.545, 95%CI: -78.620 to -28.470, *p* < 0.01). The rate of change in outpatient drug combination decelerated (0.320, 95%CI: 0.149 to 0.491, *p* < 0.01). The ratio of infusion declined for both outpatient and inpatient cases (-0.107, 95%CI: -0.209 to -0.004, *p* < 0.1; -0.843, 95%CI: -1.154 to -0.532, *p* < 0.01). However, the results revealed that overall medical cost per visit and drug proportion for outpatient care continued their initial upward trend. After the reform, the decline of drug proportion for outpatient care was less pronounced compared to the period prior to the reform, and length of stay also had a similar trend.

**Conclusion:**

To some extent, capitation under the county medical community encourages physicians to control the cost and adopt a more standardized diagnosis and treatment behavior. This study provides evidence to consider the impact of policy changes on physicians’ behavior when designing payment methods and healthcare systems aimed at promoting PHC.

**Supplementary Information:**

The online version contains supplementary material available at 10.1186/s12889-024-18411-2.

## Background

The intricate healthcare system in China has posed considerable challenges to healthcare reform over the years. The momentum of reform needs to be sustained, with particular emphasis on the enhancement of grassroots healthcare [1]. Dating back to the 1950s, China established a health service delivery network at the county, township and village level, effectively catering to rural healthcare needs and advancing universal health coverage [2]. Township hospitals served as the hub has been praised by the World Health Organization (WHO) as one of the “three magic weapons” for achieving primary healthcare (PHC) in rural China [3].

However, during the 1980s, economic system reforms led to the devaluation of the health system. This was followed by many subsequent changes, including the transfer of patients to tertiary hospitals, the downsizing of county hospitals and township hospitals, the withdrawal of prevention programs, and the dismantling of the cooperative healthcare system [4]. These transformations, chiefly attributed to the inefficiency of the healthcare system and financing systems, resulted in a squandering of medical resources, decreased healthcare efficiency, weakened grassroots capabilities, and the prevalence of the “hardship and expense in accessing healthcare” phenomenon.

Resolving these issues is a current imperative for China’s healthcare agenda. We urgently need to optimize the PHC system in rural areas and establish an efficient and collaborative healthcare system and financing system. Integrated Healthcare Delivery System (IHDS) aligns with international advocation. The development of “integrated, safe, people-oriented” by IHDS means a renewed emphasis on the significance of PHC [5], consistent with China’s “strong grassroots” strategy. Regional medical community is the practice of IHDS. The working guideline issued by The General Office of the State Council in 2015 emphasized the priority of community-level work and encouraged the establishment of medical community to improve grassroots service ability, and in 2017 formally proposed a comprehensive medical community, including the construction of county medical community [6, 7]. Moreover, payment methods are also an important component of the development of PHC and act as incentives to develop an IHDS. Without the support of payment mechanisms, progress in PHC and the requisite health service delivery systems would be hampered [8]. Therefore, it is necessary to invest substantial time and money to explore effective payment methods to support PHC in our country [8].

In China, F County carried out reform by building a county medical community with the idea of IHDS, hoping to achieve the goals of improving grassroots healthcare capabilities, basically solving the problem of medical treatment within the county, and reigning in the unreasonable escalation of medical cost [9]. Capitation is a common model of medical insurance payment in county medical community in China. Different from the other popular payment methods, which take diseases as the payment unit, capitation payment takes expense standards per capita as the payment unit, ensuring flexibility and safeguarding physicians’ income against fluctuations in service volumes, making it resilient to potential public health emergencies such as pandemics in the future [10]. This payment method has been successfully implemented in the United States, the United Kingdom, the Netherlands and other countries for over a decade, primarily in the context of PHC for general practitioners. In China, as the main force in the development of PHC, primary healthcare institutions in county areas are mainly responsible for the diagnosis and treatment of common ailments, with family physicians practiced as health gatekeepers, delivering full-cycle health management services for the signed population, and promote the realization of the hierarchical healthcare system. This payment method can promote the registration of family physicians and guide residents to seek healthcare at the grassroots level within the county [11, 12]. Therefore, the exploration of capitation is carried out in county medical community. In light of the ongoing construction of the regional medical community, corresponding organizational adjustments have occurred in healthcare institutions. The organizational structure, culture and incentive mechanism of the medical institutions have also evolved, leading to the change in human resources, which ultimately manifested in physicians’ behavior [13].

Physicians play an important role in health service delivery, with a strong professional and interest orientation [14]. Incentive mechanisms are required to guide rational diagnosis and treatment practices among physicians. In the modern medical insurance system, provider payment methods exert a direct or indirect influence on the conduct of healthcare providers and the quality of healthcare [14, 15]. From this point of view, it is necessary to consider how physicians respond to incentives in order to improve payment method design [16]. As a healthcare provider, physicians often make choices on behalf of patients and to a large extent lead the treatment of patients, resulting in different medical expenses. Their decision making is an important factor affecting the quantity of healthcare, treatment methods and costs [17]. Under the background of county medical community, physicians’ behavior will not only affect medical cost control, but also affect the operation and development of medical community. Through the reform of payment method of county medical community, physicians’ behavior and resource allocation are adjusted, promoting the implementation of hierarchical healthcare system and grassroots service capacity [18].

In this study, one of the most common chronic diseases, hypertension, was selected as an example to examine the effects of the capitation payment model within the county medical community. Hypertension is a risk factor for many diseases, but some studies have shown that the management of hypertension is inadequate in PHC in China [19, 20]. Furthermore, the reimbursement of the New Rural Cooperative Medical System disproportionately shares the burden of major diseases, with limited compensation for chronic diseases characterized by extended treatment durations and high costs. This places a considerable economic strain on patients. But the management of people with chronic diseases has potential economic benefits [21]. The document of The General Office of the State Council outlines the path for implementing capitation payment in primary healthcare institutions for chronic diseases, and the management rate of hypertension and diabetes served as key assessment criteria for the compact county medical community [22]. From this point of view, whether the healthcare system can promote the management of chronic diseases by healthcare providers is also one of the key points of policy making.

To sum up, this paper selected hypertension as a representative disease for research, mining capitation pilot data under the background of county medical community, using the interrupted time series (ITS) analysis method for the changes in physicians’ behavior (as providers of both healthcare system and medical insurance system) before and after the pilot reform, and providing the foundation for the selection of payment for PHC.

## Methods and data

### Data source

F county in a central province in China was once a national-level poverty-stricken county. In 2014, the outpatient utilization rate in the county was less than 60%, while the percentage of poverty-stricken patients reached 60%. With the construction of the county medical community as the starting point, combined with capitation payment, F County had become a typical case for exploring the IHDS and payment.

**Fig. 1:**
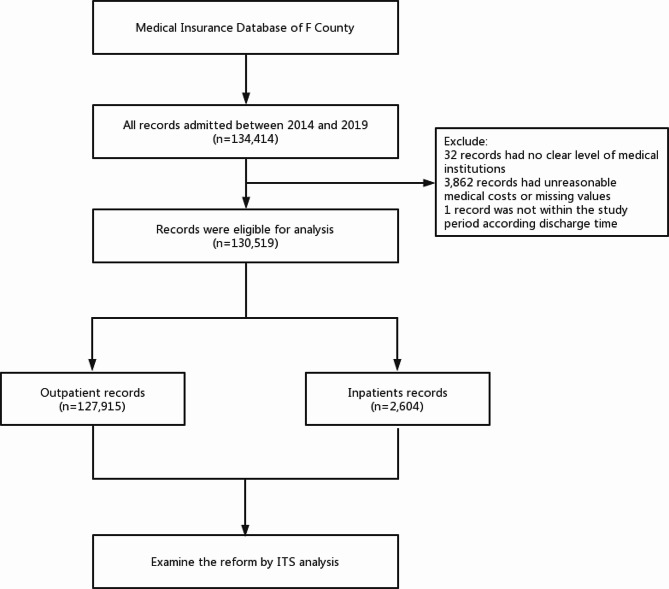
Flow chart of the study records. ITS, Interrupted time series

This county medical community initiated the reform in three batches. The first batch of six towns integrated into the county medical community in April 2015, commencing a five-year reform journey. We selected these six towns as study samples, and the data source of this paper is the New Rural Cooperative Medical Scheme database in F county. According to the township code and discharge time, the outpatient and hospitalization records of these 6 towns from 2014 to 2019 were retrieved. After extracting all the records of hypertension diagnosis and treatment in these 6 towns with a discharge diagnosis of “hypertension”, the corresponding cost detail records were matched according to the medical ID and reimbursement number. We judged the prescription of hypertension drugs according to the classification of hypertension drugs in supplemental material. A total of 134,414 records of hypertension diagnosis and prescription with at least one hypertension drug were received from the database. After screening, 130,519 records were retained, including 127,915 outpatient records and 2,604 inpatient records (Fig. 1). Monthly data before and after April 2015 (intervention point) were collected, and ITS analysis was used to obtain the changes in each outcome indicator from January 2014 to December 2019.

### Outcome variables

The following information was contained in the dataset: patient gender, age, name and level of medical institution, admission and discharge time, main diagnosis, purchased service items and specifications, medical expenses and other information.

Based on the above data information, we examined 5 variables at different levels in this study, namely, total medical cost per visit, drug proportion, length of stay, ratio of infusion and ratio of drug combination for overall, village clinics and township hospitals levels. See supplementary material for the selection and calculation of the outcome variables. Since the number of hospitalized patients with hypertension is not very large and the number of patients in different levels of medical institutions is limited, we couldn’t calculate the indicators for different medical institutions. Based on the time of discharge, we determined which month the records belonged to.

### Statistical analysis

In the realm of health policy, the implementation of policies is often not random, which requires a more appropriate research design. Interrupted time series (ITS) was first applied to health in 1981 and is now a commonly used method in the field of health policy and a strong quasi-experimental design [23]. At the fixed intervals (weekly, monthly, quarterly……) before and after the implementation of the policy, we can collect the outcome indicators that we want to analyze and use the data before the intervention to estimate the underlying long-term trend. When the counterfactual is correctly estimated, the level and slope changes resulting from the implementation of the intervention can be obtained, and the actual measurement results are compared to reflect the impact of the intervention. Changes in level and slope are often used to reflect the impact of an intervention [24].

In our study, we used Model 1 below to assess level and trend changes before and after intervention, *Y*_*t*_ is the monthly outcome variable between January 2014 and December 2019 in our study. *T*_*t*_ is the time variable (January 2014 = 0, February 2014 = 1,…, December 2019 = 71). *X*_*t*_ is a dummy variable indicating the intervention, which is denoted as 0 before April 2015, otherwise, *X*_*t*_ is denoted as 1. *X*_*t*_*T*_*t*_ is the interaction term, calculated by (*Tt* − 15) * *X*_*t*_, which is 0 before April 2015, and since April 2015, it has successively increased from zero to another (0 = April 2015, 1 = May 2015, 2 = June 2015, 3 = July 2015,…). See supplemental material.

As for the model parameters in Model 1, *β*_*0*_ estimates the baseline level of the outcome; *β*_*1*_ estimates the pre-intervention slope; *β*_*2*_ estimates the level change at the intervention point; *β*_*3*_ estimates the post-intervention slope change compared to pre-intervention.

1 $$Y_t = \beta_0 + \beta_1 T_t + \beta_2 X_t + \beta_3 X_t T_t + \epsilon_t $$

We calculated a monthly value of outcome variables, and we used segmented linear regression to evaluate the effect of single intervention. Since autocorrelation leads to spuriously small standard errors, Durbin-Watson statistic was used to detect autocorrelation, and Newey-West standard errors were used to correct autocorrelation [24, 25]. Our examination of the scatterplots showed no significant seasonality. Stata 17.0 software (Stata Corp LP, College Station, TX, USA) was used for ITS.

## Results

### Basic information on hypertension outpatients from 2014 to 2019

From 2014 to 2019, the number of hypertension outpatients exhibited a consistent annual increase. In terms of gender, there was little difference between males and females. In terms of age distribution, patients were mainly concentrated in 45–59 and 60–74 age groups, and middle-aged and elderly patients were the majority. The majority of patients belonged to the non-poor category, with little fluctuation. As can be seen from the hypertension outpatient flow, about 80% of outpatients sought medical treatment in village clinics, which was the lowest in 2019, accounting for 72.79%. Over the years, there was a progressive increase in the proportion of patients seeking healthcare at township hospitals, while those visiting county hospitals remained under 1%. Total medical cost per visit and drug proportion increased, while the ratio of infusion and the ratio of hypertension drug combination exhibited a decreasing trend. See Table 1 for details.

**Table 1:** Characteristics of outpatient hypertension visits from 2014 to 2019

	2014	2015	2016	2017	2018	2019
Sex, n (%)						
Male	7230 (42.41)	8192 (44.87)	8837 (47.30)	11,616 (48.96)	12,653 (49.33)	11,855 (48.30)
Female	7210 (42.29)	8520 (46.66)	9115 (48.79)	12,109 (51.04)	12,997 (50.67)	12,627 (51.44)
Missing	2609 (15.30)	1547 (8.47)	732 (3.91)	0 (0.00)	1 (0.00)	65 (0.26)
Age group, n (%)						
-17	14 (0.08)	5 (0.03)	6 (0.03)	30 (0.13)	16 (0.06)	14 (0.06)
18–44	1368 (8.02)	1273 (6.97)	1296 (6.94)	1626 (6.85)	1527 (5.95)	1370 (5.58)
45–59	4679 (27.44)	5270 (28.86)	5569 (29.81)	7495 (31.59)	8586 (33.47)	8801 (35.85)
60–74	6440 (37.77)	7581 (41.52)	8293 (44.39)	10,544 (44.44)	10,521 (41.02)	9567 (38.97)
75-	1935 (11.35)	2573 (14.09)	2785 (14.91)	4030 (16.99)	4999 (19.49)	4603 (18.75)
Missing	2613 (15.34)	1557 (8.53)	735 (3.92)	0 (0.00)	2 (0.01)	192 (0.79)
Poverty or not, n (%)						
Yes	3736 (21.91)	4362 (23.89)	4767 (25.51)	7578 (31.94)	8000 (31.19)	5976 (24.35)
NO	13,313 (78.09)	13,897 (76.11)	13,917 (74.49)	16,147 (68.06)	17,651 (68.81)	18,571 (75.65)
Distribution of patients, n (%)						
Village clinic	14,163 (83.07)	15,107 (82.74)	15,724 (84.16)	19,172 (80.81)	19,342 (75.40)	17,867 (72.79)
Township hospitals	2823 (16.56)	3086 (16.90)	2895 (15.49)	4516 (19.03)	6304 (24.58)	6669 (27.17)
County hospitals	63 (0.37)	66 (0.36)	65 (0.35)	37 (0.16)	5 (0.02)	11 (0.04)
Medical cost per visit, mean±(SD)	25.71± 14.73	27.80 ± 16.56	28.89 ± 16.45	32.55 ± 18.11	34.70 ± 18.14	35.05 ± 18.61
Drug proportion, %	74.50	76.07	76.81	80.38	81.06	81.11
Ratio of infusion, %	7.37	6.56	4.47	3.01	1.73	1.47
Ratio of drug combination, %	24.08	20.27	19.81	17.26	16.76	14.66
Overall	17,049	18,259	18,684	23,725	25,651	24,547

SD, standard deviation

### Changes in physicians’ behavior for outpatients with hypertension

**Fig. 2:**
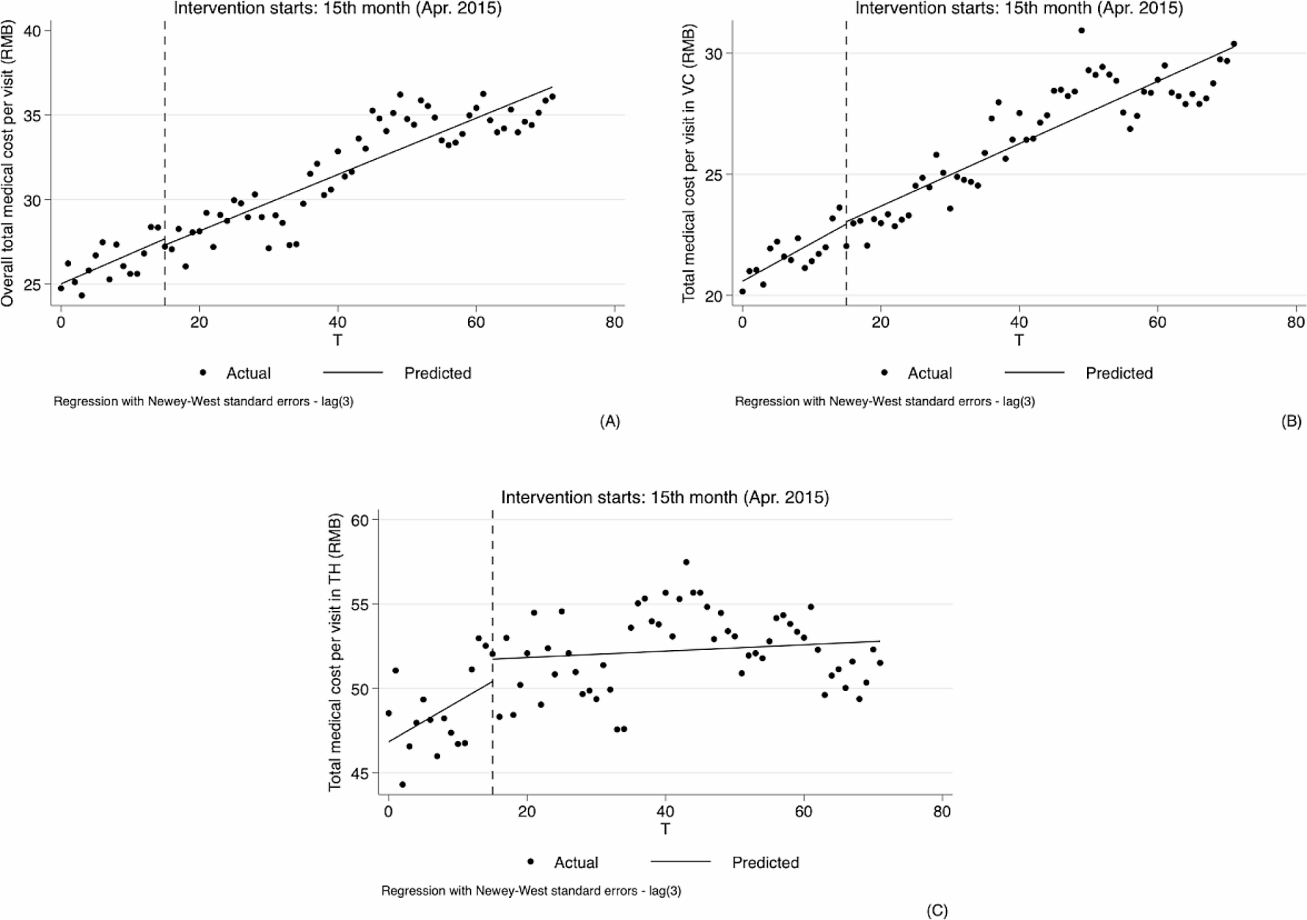
Segmented regression models showing total medical cost per visit foroutpatient before and after reform. Note: **A** Overall total medical cost per visit (RMB); **B** Total medical cost per visit in village clinics (RMB); **C** Total medical cost per visit in township hospitals (RMB). VC Village clinics, TH Township hospitals

**Fig. 3:**
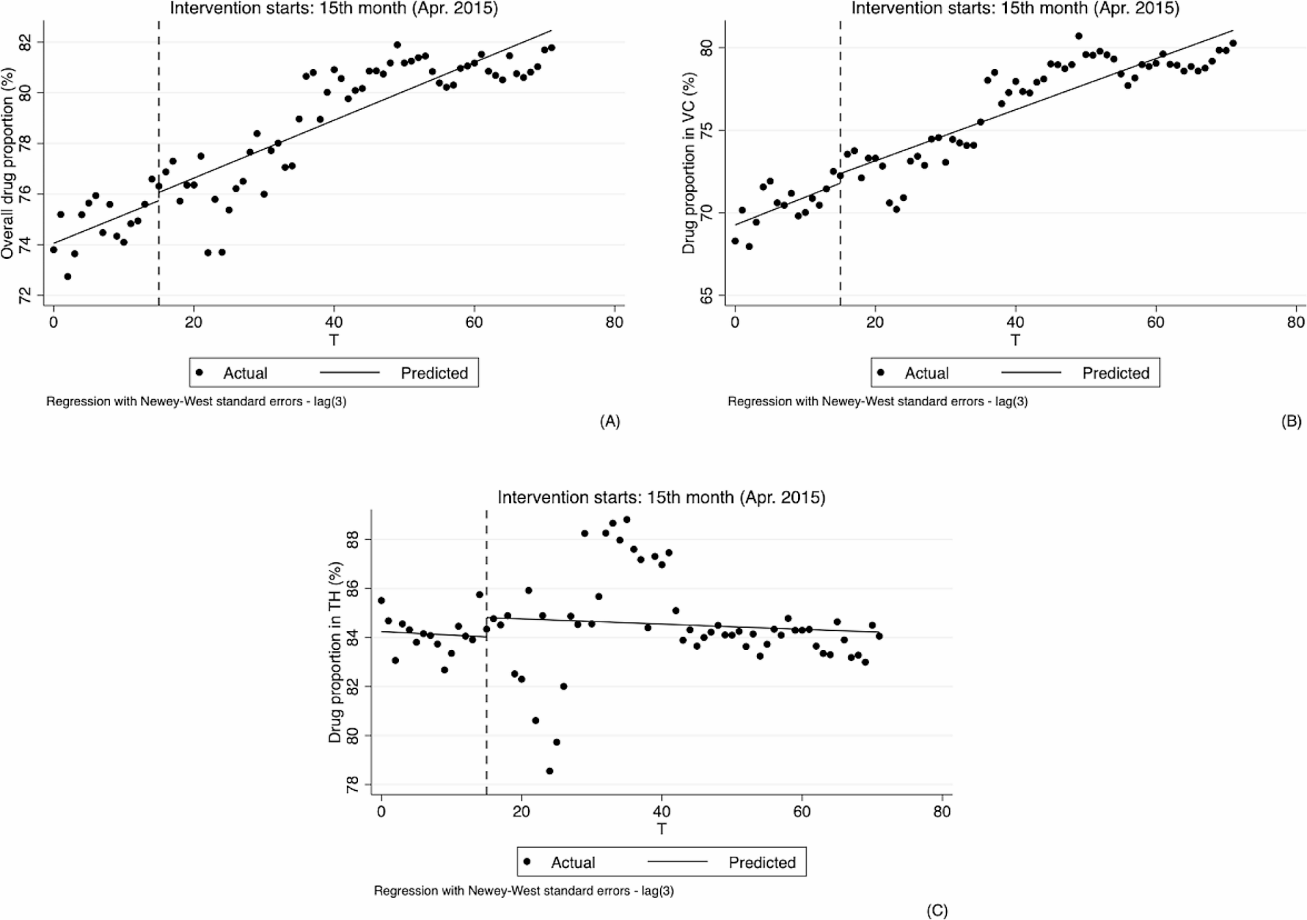
Segmented regression models showing ratio of drug combination for outpatient before and after reform. Note: **A** Overall ratio of drug combination (%); **B** Ratio of drug combination in village clinics (%); **C** Ratio of drug combination in township hospitals (%). VC Village clinics, TH Township hospitals

**Fig. 4:**
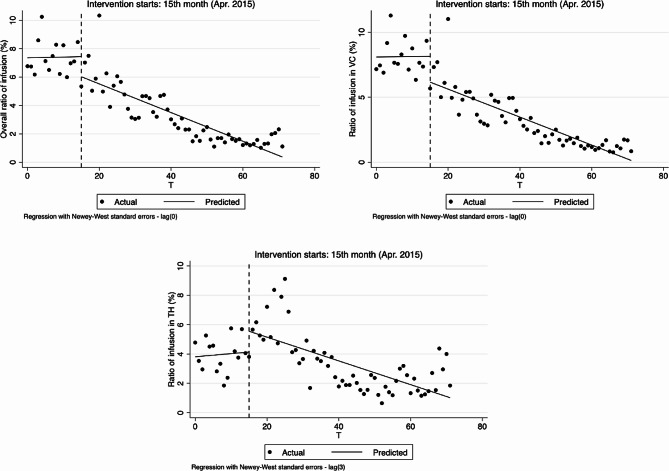
Segmented regression models showing drug proportion for outpatient before and after reform. Note: **A** Overall drug proportion (%); **B** Drug proportion in village clinics (%); **C** Drug proportion in township hospitals (%). VC Village clinics, TH Township hospitals

**Fig. 5:**
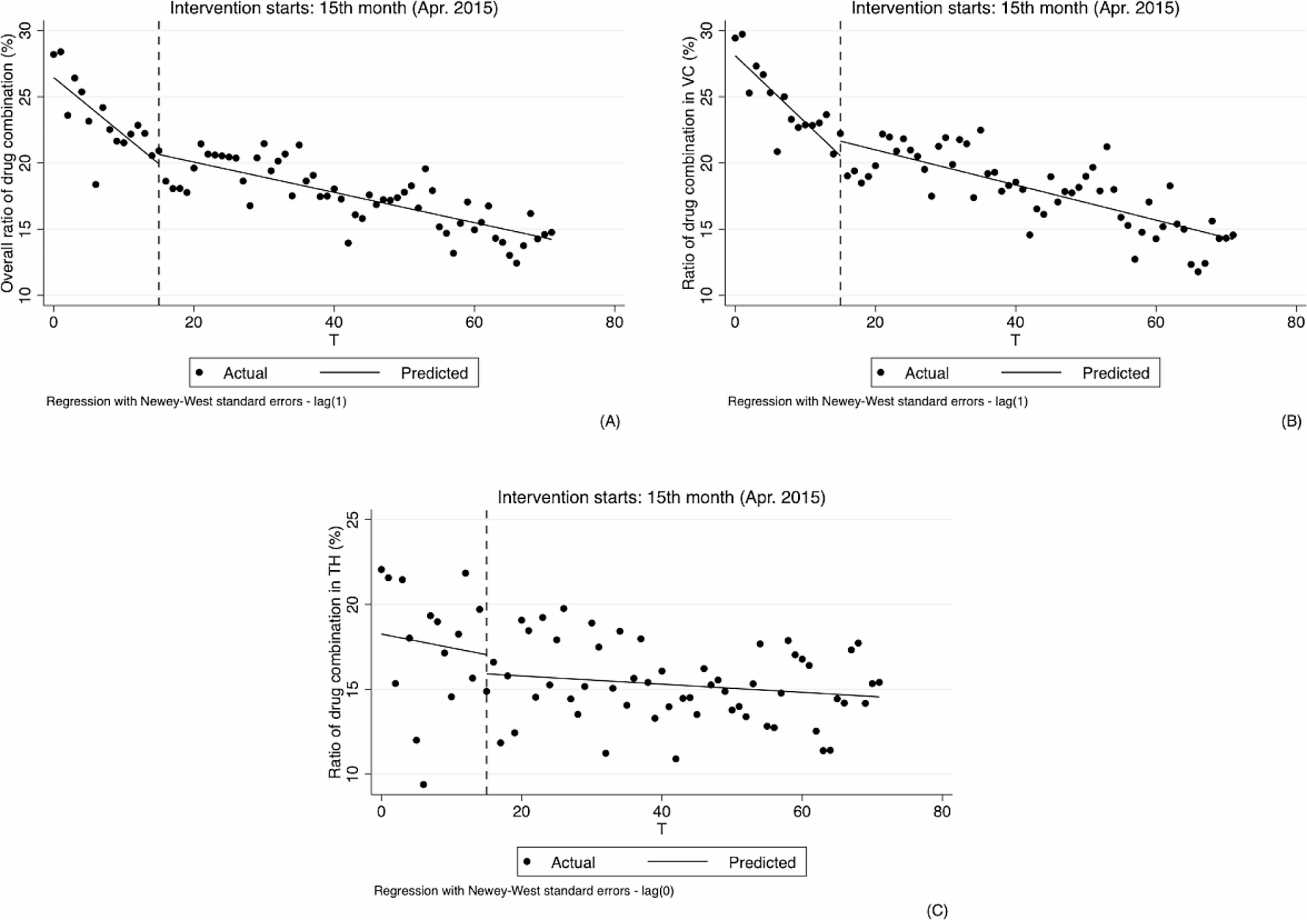
Segmented regression models showing ratio of infusion for outpatient before and after reform. Note: **A** Overall ratio of infusion (%); **B** Ratio of infusion in village clinics (%); **C** Ratio of infusion in township hospitals (%). VC Village clinics, TH Township hospitals

As for outpatient, the results of analyzing the changes in physicians’ behavior in hypertension treatment by using ITS analysis method are shown in Table 2; Figs. 2, 3, 4 and 5.

**Table 2:** Estimated changes in level and trend of outpatient indicators

	Baseline level β_0_, (95%CI)	Baseline slope β_1_, (95%CI)	Level change β_2_, (95%CI)	Slope change β_3_, (95%CI)
Medical cost per visit (RMB)				
Overall^#^	25.011*** (24.592 to 25.431)	0.178*** (0.099 to 0.256)	-0.370 (-1.517 to 0.777)	-0.011 (-0.092 to 0.071)
Village clinic^#^	20.585*** (20.240 to 20.930)	0.157*** (0.098 to 0.216)	0.096 (-0.840 to 1.033)	-0.028 (-0.089 to 0.032)
Township hospitals^#^	46.841*** (45.134 to 48.548)	0.238 (-0.027 to 0.503)	1.325 (-1.742 to 4.393)	-0.219 (-0.485 to 0.047)
Drug proportion (%)				
Overall^#^	74.056*** (73.512 to 74.600)	0.112*** (0.046 to 0.179)	0.323 (-0.777 to 1.423)	0.002 (-0.071 to 0.075)
Village clinic^#^	69.267*** (68.446 to 70.088)	0.169*** (0.085 to 0.252)	0.582 (-0.642 to 1.805)	-0.014 (-0.104 to 0.076)
Township hospitals^#^	84.240*** (83.616 to 84.864)	-0.014 (-0.108 to 0.080)	0.786 (-1.161 to 2.732)	0.004 (-0.097 to 0.104)
Ratio of infusion (%)				
Overall	7.356*** (6.383 to 8.330)	0.006 (-0.095 to 0.106)	-1.415** (-2.455 to -0.375)	-0.107* (-0.209 to -0.004)
Village clinic	8.101*** (7.024 to 9.179)	0.002 (-0.115 to 0.120)	-1.976*** (-3.188 to -0.765)	-0.110 (-0.228 to 0.008)
Township hospitals^#^	3.811*** (3.219 to 4.402)	0.021 (-0.054 to 0.097)	1.418 (-0.029 to 2.865)	-0.102** (-0.183 to -0.021)
Ratio of drug combination (%)				
Overall^#^	26.447*** (24.784 to 28.110)	-0.434*** (-0.603 to -0.265)	0.698 (-0.898 to 2.294)	0.320*** (0.149 to 0.491)
Village clinic^#^	28.097*** (26.766 to 29.429)	-0.504*** (-0.650 to -0.357)	1.106 (-0.430 to 2.643)	0.371*** (0.222 to 0.521)
Township hospitals	18.255*** (15.170 to 21.340)	-0.081 (-0.402 to 0.239)	-1.129 (-3.962 to 1.705)	0.057 (-0.265 to 0.379)

*p < 0.1, **p < 0.05, ***p < 0.01. ^#^, autocorrelation was performed. CI, confidence interval

As shown in Table 2; Fig. 2, our model found remarkable upward trends before the implementation of the reform (0.178, 95%CI: 0.099 to 0.256, *p* < 0.01; 0.157, 95%CI: 0.098 to 0.216, *p* < 0.01) at both overall and village levels, while the change in the level and trend after the implementation of the reform was not significant. In the township model, both level and slope change parameters were not statistically significant.

Similarly, at overall and village levels, the segmented linear regression analysis results indicate that the trend of drug proportion significantly rose (0.112, 95%CI: 0.046 to 0.179, *p* < 0.01; 0.169, 95%CI: 0.085 to 0.252, *p* < 0.01). After the implementation of the reform, the slightly upward trend remained unchanged, and changes in both level and slope were not statistically significant. See Table 2; Fig. 3.

Ratio of infusion at the overall, village and township levels did not increase significantly before the implementation of reform, but after the implementation of reform, at the overall and village levels, decreased by 1.415% and 1.976%, respectively (-1.415, 95%CI: -2.455 to -0.375, *p* < 0.01; -1.976, 95%CI: -3.188 to -0.765, *p* < 0.01). Compared with pre-reform, there was a significant difference in the trend of overall infusion ratio after the reform, with an average monthly decrease of 0.101% (-0.107, 95%CI: -0.209 to -0.004, *p* < 0.1, 0.006–0.107 = -0.101), the ratio of infusion at village level also showed a decreasing trend but had no statistical significance (-0.110, 95%CI: -0.228 to 0.008, *p* > 0.1), and the ratio of infusion at township level decreased by 0.102% (-0.102, 95%CI: -0.183 to -0.021, *p* < 0.05) after the implementation of the reform compared with pre-reform, equating to a monthly decrease of 0.081% (0.021–0.102 = -0.081). See Table 2; Fig. 4.

From the results of segmented linear regression analysis, ratio of drug combination at the overall and village levels showed a decreasing trend before the implementation of reform (-0.434, 95%CI: -0.603 to -0.265, *p* < 0.01; -0.504, 95%CI: -0.650 to -0.357, *p* < 0.01). While the changes in instantaneous level were not obvious after the implementation of the reform, the decline of trends were obviously slowed down, 0.114% per month (0.320, 95%CI: 0.149 to 0.491, *p* < 0.01, -0.434 + 0.320 = -0.114) and 0.133% per month (0.371, 95%CI: 0.222 to 0.521, *p* < 0.01, -0.504 + 0.371 = -0.133), respectively. In the township hospitals, the trend was not obvious before and after the reform. See Table 2; Fig. 5.

### Basic information on hypertension inpatients from 2014 to 2019

From 2014 to 2019, the number of hypertension inpatients showed a consistent decreasing trend. Similar to the outpatient department, the proportion of men and women existed little disparity, the majority of inpatients were 45–59 years old and 60–74 years old, the non-poor population was larger than the poor population, and the ratio of infusion was much larger than the outpatient department, but all showed a downward trend. On the contrary, the proportion of inpatients going to township hospitals decreased year by year, but the proportion of inpatients outside the county shows the opposite trend, Overall, drug proportion and total medical cost per visit decreased. There were no conspicuous trends in length of stay and the ratio of infusion (Table 3).

**Table 3:** Characteristics of inpatient hypertension visits from 2014 to 2019

	2014	2015	2016	2017	2018	2019
Sex, n (%)						
Male	353 (37.43)	229 (42.80)	214 (44.12)	111 (47.23)	94 (51.65)	107 (47.77)
Female	437 (46.34)	235 (43.93)	244 (50.31)	124 (52.77)	88 (48.35)	115 (51.34)
Missing	153 (16.23)	71 (13.27)	27 (5.57)	0 (0.00)	0 (0.00)	2 (0.89)
Age group, n (%)						
18–44	20 (2.12)	28 (5.23)	18 (3.71)	13 (5.53)	19 (10.44)	16 (7.14)
45–59	194 (20.57)	107 (20.00)	98 (20.21)	74 (31.49)	51 (28.02)	51 (22.77)
60–74	443 (46.98)	249 (46.54)	239 (49.28)	107 (45.53)	89 (48.90)	103 (45.98)
75-	133 (14.10)	80 (14.95)	103 (21.24)	41 (17.45)	23 (12.64)	50 (22.32)
Missing	153 (16.23)	71 (13.28)	27 (5.56)	0 (0.00)	0 (0.00)	4 (1.79)
Poverty or not, n (%)						
Yes	299 (31.71)	169 (31.59)	158 (32.58)	82 (34.89)	69 (37.91)	99 (44.20)
NO	644 (68.29)	366 (68.41)	327 (67.42)	153 (65.11)	113 (62.09)	125 (55.80)
Distribution of patients, n (%)						
Township hospitals	650 (68.93)	298 (55.70)	296 (61.03)	125 (53.19)	65 (35.71)	86 (38.39)
County hospitals	222 (23.54)	184 (34.39)	124 (25.57)	51 (21.70)	48 (26.37)	52 (23.22)
Outside the county	71 (7.53)	53 (9.91)	65 (13.40)	59 (25.11)	69 (37.92)	86 (38.39)
Medical cost per visit, mean±(SD)	2122.23 ± 1657.32	2726.82 ± 2540.39	2681.12 ± 2414.48	2675.16 ± 2516.69	2140.45 ± 1916.27	2151.96 ± 2639.43
Drug proportion, %	62.41	53.80	47.98	38.49	43.17	40.77
Length of stay, mean±(SD)	7.02 ± 3.67	7.79 ± 9.02	7.44 ± 6.82	8.34 ± 20.00	6.59 ± 17.01	7.68 ± 19.63
Ratio of infusion, %	96.82	97.20	93.61	87.23	65.38	54.91
Ratio of drug combination, %	43.80	39.63	47.84	49.79	45.60	44.20
Overall	943	535	485	235	182	224

SD, standard deviation

### Changes in physicians’ behavior for inpatient with hypertension

**Fig. 6:**
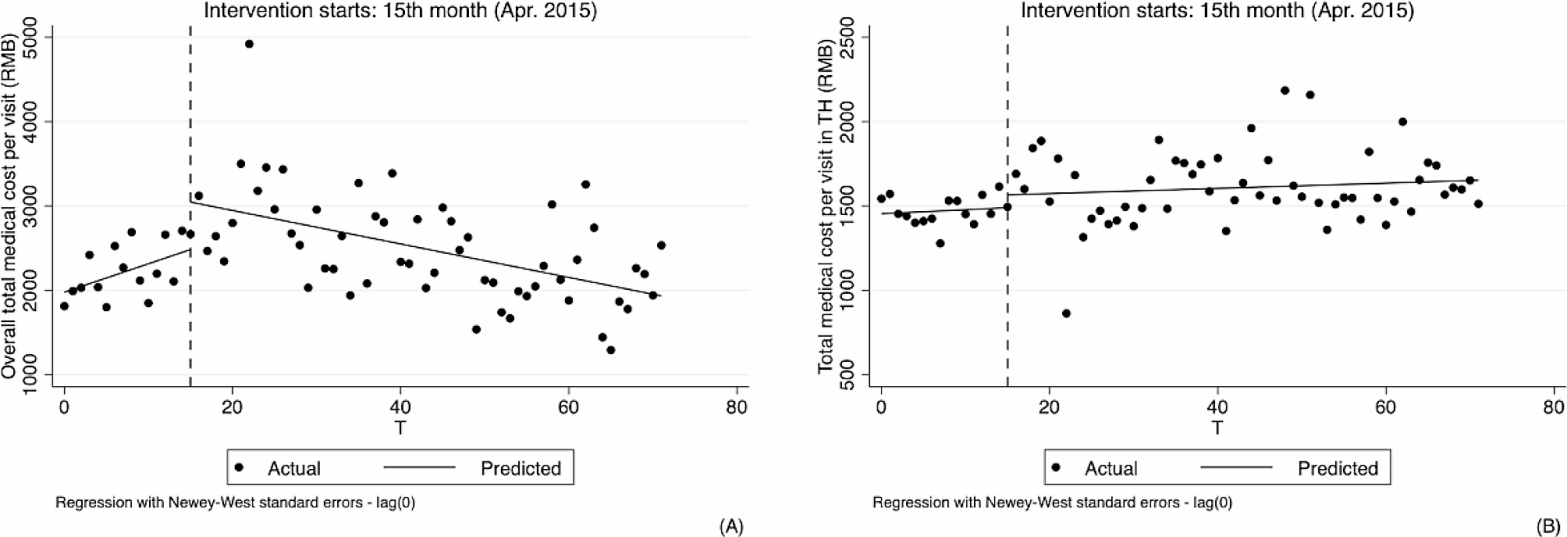
Segmented regression models showing total medical cost per visit for inpatients before and after reform. Note: **A** Overall total medical cost per visit (RMB); **B** Total medical cost per visit in township hospitals (RMB). TH Township hospitals

**Fig. 7:**
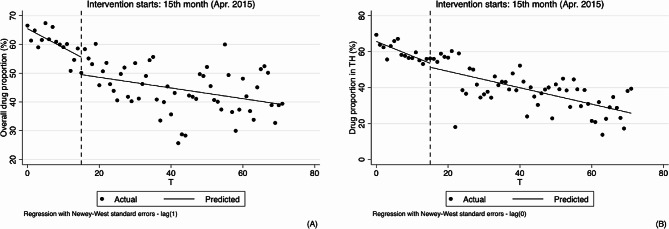
Segmented regression models showing drug proportion for inpatient before and after reform. Note: **A** Overall drug proportion (%); **B** Drug proportion in township hospitals (%). TH Township hospitals

**Fig. 8:**
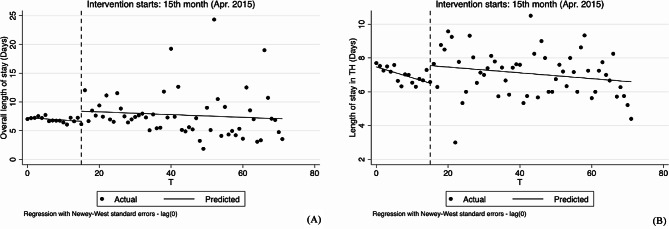
Segmented regression models showing length of stay for inpatient before and after reform. Note: **A** Overall length of stay (Days); **B** Length of stay in township hospitals (Days). TH Township hospitals

**Fig. 9:**
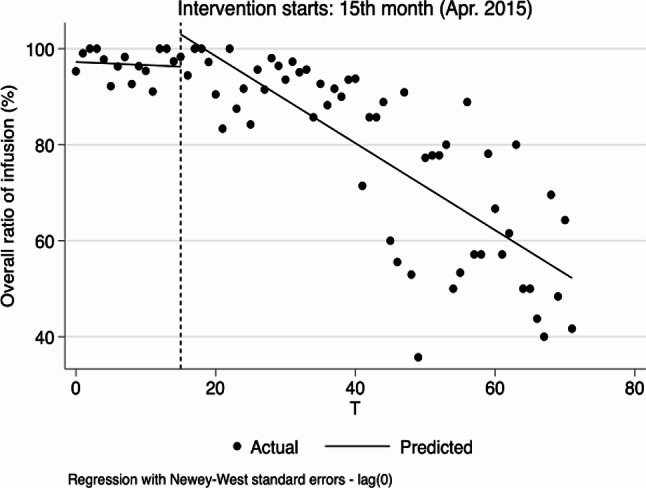
Segmented regression models showing ratio of drug combination for inpatient before and after reform

**Fig. 10:**
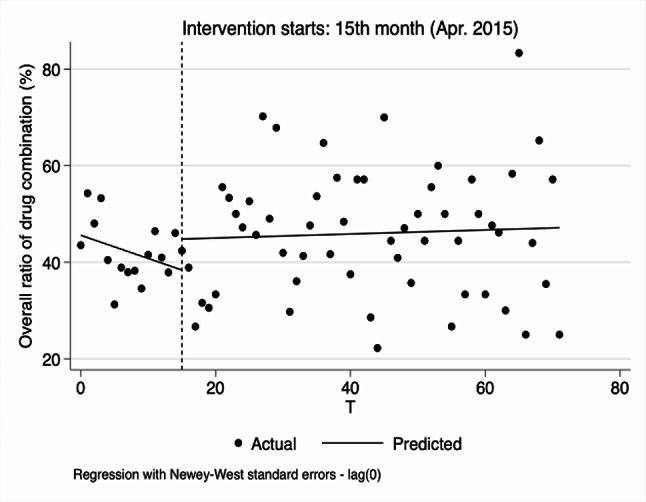
Segmented regression models showing ratio of infusion for inpatient before and after reform

The results of the changes in the treatment of hypertension in the inpatient section are shown in Table 4 Figs. 6, 7, 8, 9 and 10. Due to the small number of patients hospitalized for hypertension, some outcome measures cannot be analyzed at different levels.

**Table 4:** Estimated changes in level and slope of inpatient indicators

	Baseline level β_0_, (95%CI)	Baseline slope β_1_, (95%CI)	Level change β_2_, (95%CI)	slope change β_3_, (95%CI)
Medical cost per visit (RMB)				
Overall	1978.034*** (1809.094 to 2146.975)	33.687** (9.866 to 57.508)	562.766** (188.009 to 937.523)	-53.545*** (-78.620 to -28.470)
Township hospitals	1454.109*** (1384.741 to 1523.478)	2.362 (-6.461 to 11.185)	75.973 (-54.540 to 206.486)	-0.809 (-10.055 to 8.437)
Drug proportion (%)				
Overall^#^	65.481*** (63.268 to 67.695)	-0.661*** (-0.956 to -0.366)	-6.065** (-10.486 to -1.644)	0.475** (0.161 to 0.789)
Township hospitals	65.679*** (62.590 to 68.768)	-0.811*** (-1.103 to -0.519)	-2.192 (-7.012 to 2.629)	0.355* (0.035 to 0.675)
Length of stay (Days)				
Overall	7.261*** (7.010 to 7.511)	-0.042* (-0.081 to -0.003)	1.767** (0.561 to 2.972)	0.018 (-0.044 to 0.080)
Township hospitals	7.472*** (7.242 to 7.702)	-0.064*** (-0.100 to -0.028)	1.040** (0.251 to 1.829)	0.047* (0.006 to 0.088)
Ratio of infusion (%)				
Overall	97.226*** (95.143 to 99.309)	-0.064 (-0.344 to 0.217)	6.719** (2.284 to 11.154)	-0.843*** (-1.154 to -0.532)
Ratio of drug combination (%)				
Overall	45.573*** (40.147 to 50.999)	-0.479 (-1.060 to 0.102)	6.420 (-0.791 to 13.632)	0.521 (-0.091 to 1.134)

*p < 0.1, **p < 0.05, ***p < 0.01. #, autocorrelation was performed. CI, confidence interval

At the overall level, the results showed that a consistent upward trend was apparent in the total medical cost per visit before the reform (33.687, 95%CI: 9.866 to 57.508, *p* < 0.05), and instantaneously increased by 562.766 yuan after the reform (562.766, 95%CI: 188.009 to 937.523, *p* < 0.05). However, an opposing trend was noted after the reform (-53.545, 95%CI: -78.620 to -28.470, *p* < 0.01), with a monthly decrease of 19.858 yuan (33.687–53.545 = -19.858). Total medical cost per visit in township hospitals showed no obvious trend before and after the reform (Table 4; Fig. 6).

The overall drug proportion decreased by 0.661% per month before the reform (-0.661, 95%CI: -0.956 to -0.366, *p* < 0.01), and immediately decreased by 6.065% after the reform (-6.065, 95%CI: -10.486 to -1.644, *p* < 0.05). The downward trend after the reform was significantly slower than that before the reform (0.475, 95%CI: 0.161 to 0.789, *p* < 0.05), with an average monthly decline of 0.186% (-0.661 + 0.475 = -0.186). At the township level, drug proportion showed similar level and slope changes before and after the reform. Before the reform, it decreased by 0.811% per month (-0.811, 95%CI: -1.103 to -0.519, *p* < 0.01), and in April 2015, the drug proportion decreased by 2.192% but had no statistical significance. After the reform, the drug proportion decreased by 0.456% per month (0.355, 95%CI: 0.035 to 0.675, *p* < 0.1; -0.811 + 0.355 = -0.456) (Table 4; Fig. 7).

The overall length of stay showed a slight decrease before the implementation of the reform (-0.042, 95%CI: -0.081 to -0.003, *p* < 0.1). But it immediately grew by 1.767 days in April 2015 (1.767, 95%CI: 0.561 to 2.972, *p* < 0.05), and the trend change after the reform was not obvious compared with that before the reform. At the township level, length of stay showed a decreasing trend before the reform (-0.064, 95%CI: -0.100 to -0.028, *p* < 0.01). The reform was associated with a significant increase in the level (1.040, 95%CI: 0.251 to 1.829, *p* < 0.05) and a slight increase in the slope (0.047, 95%CI: 0.006 to 0.088, *p* < 0.1) (Table 4; Fig. 8).

Before the reform, the overall trend of ratio of infusion was not obvious. However, overall ratio of infusion increased by 6.719% in the reform month (6.719, 95%CI: 2.284 to 11.154, *p* < 0.05), and the slope of that significantly decreased after the reform (-0.843, 95%CI: -1.154 to -0.532, *p* < 0.01) (Table 4; Fig. 9).

As presented in Table 4; Fig. 10, although the policy was introduced, overall ratio of drug combination did not change significantly.

## Discussion

As the literature showed, many factors have an impact on physicians’ behavior. Notably, financial incentives are found to have a more rapid response relative to factors such as professional ethics and code of conduct education on physicians’ behavior. Before the implementation of capitation reform in County F, the sample in this study mainly implemented Fee-for-Service (FFS). FFS often leads to the behaviors of over-prescription and over-examination, leading to an irrational escalation in medical costs [26]. According to the results of capitation under the county medical community, the reform has a certain impact on physicians’ behavior.

As the results of ITS showed, although total medical cost per visit of hospitalization in this study increased by 562.766 yuan instantaneously, it subsequently showed an obvious downward trend, which was similar to the results of Su [27]. The reason for the long-term downward trend in costs might be attributed to the way of the fund balance allocation within the medical community. The allocation of the health insurance fund in County F occurred quarterly to leading hospitals within the medical communities according to the number of people covered by each medical community. Any medical costs exceeding the fund wouldn’t be supplemented. If there is any fund surplus, county medical community has the right to distribute the surplus fund, including the purchase of equipment and distribution to physicians. The choice of physicians’ behavior is the result of a combination of individual physician interests, patient interests, and social interests [28]. In this study, the balance distribution in capitation was closely related to physicians’ personal interests, so it could affect physicians’ behaviors. Through the balance distribution mechanism, physicians can be encouraged to pay attention to the awareness of cost control. On the one hand, physicians proactively adopt preventive measures, bolster early disease screening and strengthen the management of chronic diseases [29], which reduce the number of hospital visits and the medical cost per visit. On the other hand, for patients who do need to be referred for treatment, physicians in a hierarchical healthcare system can follow the up-down referral process to carry out standardized diagnosis and treatment, improve service quality, reduce patients’ medical treatment for more serious diseases caused by ailment from a long-term perspective, so as to save medical costs and promote the transformation from “disease-centered” to “health-centered”.

As the surplus of the medical insurance fund can be reserved for increasing the income of physicians in the medical community [30], physicians’ practices may become more regulated. In both outpatient and inpatient parts, the results of the study showed that the proportion of infusion use decreased. Physicians should prescribe drugs for patients as much as possible, instead of infusion, which is a risky and costly method of administration. This way could not only reduce adverse drug reactions [31] but also save medical costs. Our result is similar to the results of previous research on the implementation of capitation under New Rural Cooperative Medical System in China [32]. However, in August 2014, the Former Anhui Provincial Health and Family Planning Commission Office issued a document on the management of intravenous infusion, strengthening the supervision and inspection of outpatient and emergency intravenous infusion. This directive might also have an impact on the diagnosis and treatment behavior of physicians. Regardless of the ratio of infusion, the improvement of the ratio of drug combination in the outpatient department still reflects the change in physicians’ behavior. Most patients with hypertension need to be treated with a combination of drugs. Although the ratio of drug combination in this study remained modest, the downward trend before the reform had eased after the reform. In this change, physicians, as the “agent” of patients, played a leading role in the medication of patients with hypertension. After the reform, physicians standardized prescribing, controlled the blood pressure level of patients with hypertension, and prevented the occurrence of adverse consequences [33].

In this study, we also found some problems. After the reform, total medical cost per visit and drug proportion of outpatient showed a similar upward trend as before the reform, which was similar to the results of Dong’s study [34]. In addition, although the drug proportion of inpatients instantaneously decreased by 6.065%, the rate of decline subsequently decreased compared with that before the reform. The instantaneous level of hospitalization days increased, and the rate of decline in township hospitals slowed down. Analysis of the possible reason is that physicians tried their best to prescribe drugs for hypertension treatment in the outpatient department. According to the detailed rules and regulations of F County, “50 + N” diseases shall be admitted by township hospitals. If the admission rate of township hospitals does not increase but decreases, the medical insurance fund allocated to township hospitals will be deducted proportionally, so as to guide the providers to rationally admit and transfer patients. Hypertension belongs to the category of “50 + N” diseases. The results of this study showed that the number of outpatient visits increased and the proportion of hypertension patients in township hospitals increased over the years, which was consistent with the regulations. Correspondingly, the number of inpatients decreased and the proportion of patients treated in county hospitals or outside the county increased, indicating that the original choice of hospitalization was controlled by anti-hypertensive drugs in the outpatient department as far as possible. The patients who still need hospitalization predominantly exhibit acute severe hypertension, or other diseases comorbidity. Although the LOS was prolonged and the proportion of drugs was increased, it helped mitigate the irregular transfer of patients from outpatient to inpatient status, which was in line with the reform’s purpose of saving medical resources and improving the operational efficiency of the healthcare system. In this respect, it may be more advantageous than the implementation of capitation only in the outpatient department [35].

According to the results, the outcome indicators of township hospitals changed little before and after the reform (for example, medical cost per visit, drug proportion, and ratio of drug combination after the reform showed no statistical significance in the instantaneous level and trend changes). As the hub of the three-tier health service delivery network of the county medical community, township hospitals are responsible for the diagnosis and treatment of common diseases in the county, the management of village clinics, the training of personnel, the implementation of the two-way referral system and other important work. County F had carried out the construction of county medical communities and also implemented various supporting reform measures such as reform of the capitation payment method, upgrading and renovation of hardware facilities in township health centers, superior management instructing subordinates and real-time guidance of general practitioners by specialists through information technology, with an effort to improve the service capacity and governance of PHC institutions. F County had developed clinical pathways for common diseases and provided training to strengthen the technical proficiency of personnel in primary healthcare institutions, thereby increasing the trust and satisfaction of residents in the services of primary care institutions. Patients with chronic diseases such as hypertension receive timely and effective diagnosis and treatment in township hospitals, with their daily medication needs addressed in village hospitals. Two-way referrals performed by township hospitals facilitate the compliance of function orientations, promote collaboration within the county medical community, and promote the realization of the goal of “treating major illnesses within the county, treating minor illnesses nearby, and preventing together.

### Limitation

The study also has the following limitations. First, we failed to obtain comparable township records, failed to perform multiple group ITS analyses, and did not include covariates in the model to completely eliminate other factors that influence physicians’ behavior (failing to show a significant trend over time) [36]. These factors may include physicians’ characteristics (age, job title, professional ethics, etc.) [37] and patients’ characteristics (patient financial situation, disease risk, etc.) [38] that influenced physicians’ behavior. Second, we were not able to collect changes in blood pressure of these hypertension patients before and after the reform to evaluate physicians’ behavior in terms of health outcomes. Third, indicators related to the quality of healthcare, such as mortality, hospitalization rates, and complication rates, have not been studied [39]. We could not determine whether improvements in such behavior indicators as costs have lost the quality of healthcare. Scholars can verify research on that in later studies. Fourth, the study sample area is one of the few counties in China that has implemented capitation reform for outpatient and inpatient care. Considering the slow change of physicians’ behavior, we chose the first six townships in the reform to make the time span of the data after reform as long as possible. However, the results may only be relevant for comparable regions, so the generalizability of the study is limited.

## Conclusion

In China, matching the right incentives in the health financing system can guide providers to be efficient, high-quality and economical. This paper compared the changes in indicators related to physicians’ behavior in providing treatment to hypertensive patients before and after the implementation of capitation in F County under the background of county medical community. From the perspective of service process, we analyzed that physicians reduce the use of intravenous infusion treatment and tried their best to control blood pressure of hypertension patients by prescribing drugs in a more standardized way. It saved medical cost, but also worsened the proportion of costs spent on drugs. At the same time, it was found that the performance of township hospitals was poor. While the results have been mixed, our analysis highlights the significance of evaluating physicians’ behavior of medical insurance payment method reform. Policy makers can further improve the reform of payment methods and other related supporting mechanisms.

### Electronic supplementary material

Below is the link to the electronic supplementary material.

Supplementary Material 1

## Data Availability

As the data used in our research group is confidential and we have signed a non-disclosure agreement, we regret that we cannot publicly share the original data. However, the data sets analyzed in this study are available from the corresponding author on reasonable request.
