# Supplementary material for: Impact of capitation on physicians’ behavior among patients with hypertension: an interrupted time series study in rural China
Source: BMC Public Health. 2024 May 3;24:1229. doi: 10.1186/s12889-024-18411-2 (PMC11069216; doi:10.1186/s12889-024-18411-2)
Supplement: Supplementary file 1 — Supplementary Material 1 [file 12889_2024_18411_MOESM1_ESM.docx]

**Impact of capitation on physicians’ behavior among patients with hypertension: an interrupted time series study in rural China**

Government financial subsidies to public hospitals are not enough to support hospital operations, so hospitals often obtain more medical income by increasing the provision of healthcare to make up for hospital operating expense and maintain normal operations. The reform of payment method is intended to motivate physicians to behave better and avoid increasing income through excessive treatment. Under capitation payment, hospitals remedy income without providing excessive healthcare.

Due to the information asymmetry and the monopoly of medicine, patients usually entrust physicians to decide the healthcare they need, including treatment methods and the number of services, and different treatment methods and the number of services will lead to different costs. Therefore, we choose the following indicators. (1) Medical cost per visit is the total medical cost incurred by patients. The reform of payment method can stimulate the driving forces of physicians to control cost, and restrain the cost increase caused by unreasonable treatment behavior^1^. (2) Drug proportion refers to the proportion of drug cost generated by each visit within the total cost. Effective payment method motivates physicians to regulate prescribing behavior and reduce unnecessary drug prescriptions, thus reducing the proportion of drug cost in total cost^2^. (3) Length of stay is selected to reflect service efficiency and intensity. Shortening length of stay without shrinking in quality can reduce costs and improve efficiency^3,4^. (4) Ratio of infusion refers to the ratio of infusion administration, reflecting the administration by physicians. Compared with drug prescription, infusion has higher clinical risk and higher cost. (5) The proportion of drug combination refers to the proportion of hypertension patients whose prescriptions contain two or more hypertension drugs (classification in Appendix Table 1). Most hypertension patients need to be treated with combination medication^5^. Standardized prescription can lead to better blood pressure control, less severe disease and lower cost.

Appendix Table 1 Hypertension classification and drug names in our study

| Classification | Drug names |
| --- | --- |
| Angiotensin-Converting Enzyme Inhibitor (ACEI) | Fosinopril, Benazepril, Perindopril, Enalapril, Captopril, etc |
| Angiotensin II Receptor Blocker (ARB) | Valsartan, Irbesartan, Candesartan, Olmesartan, Telmisartan, etc |
| β-blocker | Metoprolol, Bisoprolol, Propranolol, Esmolol, Timolol, Verapamil, Lacidipine, Benidipine, etc |
| Calcium Channel Blocker(CCB) | Nifedipine, Felodipine, Nirendipine, Amlodipine, Nimodipine, etc |
| Diuretic | Indapamide, Hydrochlorothiazide, Furosemide, Spirolactone, Amphenophenidine, Tolasemide, etc |
| Central acting drug | Reserpine, Clonidine, etc |
| α, β receptor antagonist | Carvedilol, Labetalol, etc |
| α receptor antagonist | Terazosin, etc |
| Polypill | Compound reserpine dipteridine, Compound Apocynum herb tablet, Valsartan/Hydrochlorothiazide, Compound dihydrazidine sulfate, Amlodipine/Benazepril, Irbesartan/Hydrochlorothiazide, Compound captopril, Amlodipine/Valsartan, Olmesartan ester/Amlodipine, Telmisartan/Hydrochlorothiazide, Perindopril/Indapamide, Shakubaqu/Valsartan, etc |
| Traditional Chinese medicine or proprietary Chinese medicine | Qiju Dihuang pill, Songling Xuenkang Capsule, Zhenju antihypertensive tablet, Bezoar antihypertensive tablet, Qingnao antihypertensive tablet, etc |

Appendix Table 2 Covariates in our ITSA

| Time | *T_t_* | *X_t_* | *X_t_T_t_* |
| --- | --- | --- | --- |
| 2014.01 | 0 | 0 | 0 |
| 2014.02 | 1 | 0 | 0 |
| 2014.03 | 2 | 0 | 0 |
| ...... | ...... | 0 | 0 |
| 2015.02 | 13 | 0 | 0 |
| 2015.03 | 14 | 0 | 0 |
| **2015.04** | 15 | 1 | 0 |
| 2015.05 | 16 | 1 | 1 |
| 2015.06 | 17 | 1 | 2 |
| ...... | ...... | 1 | ...... |
| 2019.10 | 69 | 1 | 54 |
| 2019.11 | 70 | 1 | 55 |
| 2019.12 | 71 | 1 | 56 |

**NOTE** ITSA, Interrupted time series analysis

Reference

1. Ling L Zhang. Study on the impact of medical insurance payment system reform on medical behavior. [in Chinese]. Shenyang: Shenyang Pharmaceutical University. 2021.

2. Powell-Jackson T, Yip WCM, Han W. Realigning Demand and Supply Side Incentives to Improve Primary Health Care Seeking in Rural China: IMPROVING PRIMARY HEALTH CARE SEEKING IN RURAL CHINA. Health Econ. 2015 Jun;24(6):755–72.

3. Li H, Chen Y, Gao H, Zhang Y, Chen L, Chang J, Su D, Lei S, Jiang D, Hu X. Effectiveness evaluation of quota payment for specific diseases under global budget: a typical provider payment system reform in rural China. BMC Health Serv Res. 2018 Dec;18(1):635.

4. Lai Y, Fu H, Li L, Yip W. Hospital response to a case-based payment scheme under regional global budget: The case of Guangzhou in China. Social Science & Medicine. 2022 Jan;292:114601.

5. Guidelines for Prevention and Treatment of Hypertension in China (2018 Revision). [in Chinese]. Chinese Journal of Cardiovascular Science. 2019 24(1):24–56.
